# The relationship of climate change awareness and psychopathology in persons with pre-existing mental health diagnoses

**DOI:** 10.3389/fpsyt.2023.1274523

**Published:** 2023-11-27

**Authors:** Nadja Gebhardt, Lukas Schwaab, Hans-Christoph Friederich, Christoph Nikendei

**Affiliations:** Department for General Internal Medicine and Psychosomatics, Heidelberg University Hospital, Heidelberg, Germany

**Keywords:** mental health, depression, anxiety, psychosomatic patients, climate anxiety, eco anxiety, psychoterratic syndrome

## Abstract

**Introduction:**

Persons with pre-existing mental health diagnoses are known to be more vulnerable to the consequences of climate change, such as extreme weather events and rising temperatures. However, it remains unclear if this holds true for adverse effects of climate change awareness, too.

**Methods:**

*N* = 89 patients of a psychosomatic outpatient clinic were assessed with well-established mental health questionnaires (PHQ-9 for depressive, GAD-7 for anxious, and PTSS-10 for post-traumatic symptoms) in their original form and in a modified version (PHQ-9-C, GAD-7-C, PTSS-10-C) specifically asking for patients’ symptom load regarding climate change awareness, and instruments evaluating personality factors (OPD-SF, SOC, RQ).

**Results:**

21% of the sample reported at least mild symptoms of anxiety regarding climate change awareness, and 11% mild symptoms of depression due to climate change awareness. General anxiety (GAD-7) scores significantly predicted if people reported any psychological symptoms due to climate change awareness. In multiple regression analyses, higher scores of clinical symptoms of depression, anxiety or post-traumatic stress predicted higher scores of depressive, anxious or post-traumatic symptoms regarding climate change awareness, and higher scores of psychological symptoms regarding climate change awareness predicted each other. Younger participants reported significantly more traumatic symptoms regarding climate change awareness.

**Discussion:**

The reported mental health impairments regarding climate change awareness in persons with pre-existing mental health diagnoses indicate an increased vulnerability. Hereby, depressive mental health burden seems to induce a predominantly depressive processing of climate change resulting in climate chance related depression. This holds also true for anxious and traumatic symptoms, and points toward biased attentional and memory processes and mood congruent processing.

## 1 Introduction

Anthropogenic climate change is characterized by rising average temperatures (1) and more frequent extreme weather events (2, 3). This leads to the destruction of landscapes and settlements, resulting in direct health risks for humanity: malnutrition and hunger, higher prevalences of communicable diseases, forced migration, struggle for habitats and resources, and loss of wealth (4–6). The WHO therefore identifies climate change as the greatest threat not only to human physical health, but also mental health (7). Mental health is impacted by climate change in numerous ways, directly and indirectly, on a short- and long-term basis, and these impacts are interacting (8). Overall, an increase in psychiatric disorders and suicide rates is reported to be associated with all climate change induced health risks: extreme weather events like heatwaves, floods, or wildfires, result in higher incidences of traumatic stress, general anxiety, depression, phobias, alcohol abuse, and drug impairment (9). In the long run, the ensuing destruction of landscapes and settlements is causing further adverse mental health effects through violent conflicts for resources, loss of social connection, and forced migration (4, 9). Additionally, gradual increases in average temperatures and aerosol concentrations (i.e., smoke, dust, and pollen) correlate with higher incidences of aggressive and violent behaviors, suicides, and psychiatric disorders (8, 10–12). Although an increase both in suicide rates and psychiatric disorders in response to rising average temperatures is known, there exists no literature on the interplay of those two factors.

Awareness for the existential threat posed by climate change can lead to psychological distress (13). This distress can express itself in a wide range of emotions and mental states, comprising general distress, guilt, shame, worry, anxiety, fear, phobia, paralysis, (pre-) traumatic stress, anger, melancholia, grief, or despair (8, 14). Experiencing these emotions is not inherently pathological: rather, it is also associated with desirable effects such as pro-environmental behavior (15, 16). However, psychological distress caused by climate change awareness has the potential to negatively affect mental health (17), although research remains inconclusive regarding its relationship with psychopathology (18). So far, there have been preliminary findings suggesting climate change related mental health impairments through functional impairment, symptoms of depression, anxiety, PTSD, stress, and insomnia (19, 20). As extreme emotional reactions to climate change seem to be adequate and are likely to occur, resulting serious mental health impairments through climate change awareness require professional treatment. Therefore, its impact on people whose mental health is already impaired is of great interest.

Simultaneously, psychological distress due to climate change awareness is still an emerging concept, and its definition lacks clarity (21). Two recent review articles on the mental health impacts of climate change employed the term ‘psychoterratic syndromes’ to encompass all mental health impairments related to climate change (8, 22). Following this conceptualization, we define climate change awareness related symptoms as ‘Psychoterratic SYndromes through Climate change Awareness’ (PSYCA) as the concept of interest in our study. In our understanding, this focuses on the awareness for climate change as an elicitor, but comprises a wide range of emotions as an expression of a psychoterratic syndrome. People’s understanding of climate change might vary in this concept, as it is defined by the psychological impact of such individual assessments. This is in line with the approach of previous research trying to assess the impact of climate change on mental health: in recent years, several questionnaires have been developed to assess different aspects of PSYCA (23–27). Of those, the Climate Anxiety Scale (24), measuring cognitive-emotional impairment and functional impairment through anxiety-related symptoms, has already been employed and validated in several countries (17). However, findings on its validity in replication studies were mixed (26). Furthermore, even though the original study reports correlations with a general measure of depression and anxiety (PHQ-4), and the construct has been shown to account for a significant share of the variance in symptoms of general anxious and depressive symptoms (28), it is not possible to quantify the mental health impairments assessed with the Climate Anxiety Scale in a psychopathological sense. Yet, the psychopathological aspects of PSYCA are of great interest when considering prevention and treatment options for people who are affected by it. As shown in previous studies (29, 30), adapting well-established mental health questionnaires to climate change is a valid option to assess psychopathological PSYCA symptoms. The present study followed this approach.

To explore the mental health impairments caused by PSYCA, individuals with pre-existing mental health diagnoses are a group of interest out of two reasons: firstly, persons whose mental health is already impaired might find it particularly challenging to adapt to additional climate-related stressors and might therefore present a vulnerable group to PSYCA (9). Mental health diagnoses like depression, anxiety, and post-traumatic stress disorder are associated with altered stress responses (31, 32). As a substantial part of the population reports to be worried by the existential threat posed by climate change (17, 29), those individuals among them with a mental health diagnosis might be less capable to cope with such a threat, and therefore more prone to develop psychopathological symptoms. Clearly, it is important to better understand the impairments in a group that is potentially in greater need for prevention and treatment, and for which a heightened vulnerability toward direct consequences of climate change (such as extreme weather events) has already been demonstrated (33).

Secondly, when evaluating the psychopathological aspects of PSYCA, the interrelatedness with general psychopathology is of interest to better understand which aspects of PSYCA might be linked to specific mental disorders. This information could help to tailor prevention and treatment approaches, and maybe also to understand which aspects of already existing treatment approaches can be transferred onto the treatment of PSYCA. Furthermore, when assessing PSYCA, personality factors and ontological beliefs seem to have a greater influence than demographical factors like gender, age, or social group (34). Personality factors that are known to be associated with mental health impairments in general, such as the level of structural integration (35), attachment style (36), and sense of coherence (37), might therefore also be of interest when trying to understand PSYCA. While a substantive body of research on the interplay of personality factors and pro-environmental behavior or climate change denial exists (38, 39), the authors found no conclusive literature on the relationship of personality factors and PSYCA. In a previous study with medical students conducted by our team (30), the same personality factors were associated with a measure of perceived stress when thinking about climate change; however, the absolute number of students reporting a significant anxious, depressive, or post-traumatic symptom load due to PSYCA was too small to analyze such associations. Thus, such considerations remain exploratory in nature (40). Nevertheless, other studies show more pronounced mental health impairments in younger and female participants [(17); Ogunbode et al., 2023], underlining the need to explore these factors, as well.

In synthesis, our research aimed at answering the following questions: (i) Do persons with pre-existing mental health diagnoses show a quantifiable amount of psychopathology due to PSYCA? (ii) Are psychopathological symptoms of depression, anxiety, and post-traumatic stress predicting psychopathological PSYCA symptoms in persons with pre-existing mental health diagnoses? (iii) Are personality factors or demographical factors predicting psychopathological PSYCA symptoms in persons with pre-existing mental health diagnoses?

## 2 Materials and methods

### 2.1 Study design, participants and procedure

The present study explored the data of *n* = 89 patients of a psychosomatic outpatient clinic. Participants were recruited at the Clinic for General Internal Medicine and Psychosomatics at the Heidelberg University Hospital between May and December 2021 as a convenience sample. Patients were approached when waiting for their appointments for a diagnostic interview and informed about the purpose and design of the study and signed an informed consent. They were asked to fill out an online questionnaire within 2 weeks following their appointment if they agreed to participate. Completion took approximately 20 minutes. All measures were administered in one questionnaire. If participants completed the online survey, the mental disorders diagnosed in the diagnostic interview were retrieved. Psychosomatic outpatients at the Heidelberg University Hospital were diagnosed according to the ICD-10 classification system (41) by clinical experts. The study was approved by the ethics committee of the Medical Faculty of the University of Heidelberg (S-450/2021) and was in line with the Declaration of Helsinki.

### 2.2 Measures

#### 2.2.1 Attitudes toward climate change

A prerequisite to experience PSYCA is a basic knowledge about climate change and its consequences on an individual, societal, and global level. To assess if participants were indeed aware of climate change and its consequences, ten questions of the eighth round of the European Social Survey pertaining to climate change were used (42). Questions were concerned with how participants perceived climate change and which role it played in their daily lives, the extent of their worries about climate change, and their perceived ability and responsibility to mitigate its effects.

#### 2.2.2 Measures to assess mental health impairments

As an objective criterion of psychopathology, diagnoses of mental disorders according to the ICD-10 (41), established in a diagnostical interview by trained clinicians, were retrieved. Additionally, participants’ symptom load was determined psychometrically with well-established mental health questionnaires. Depressive symptoms were measured with the Patient Health Questionnaire [PHQ-9; (43)], assessing a decrease of interest or pleasure in doing things, feeling down, depressed, or hopeless; asking for sleep, eating and concentration problems, the feeling to be a failure and to disappoint, psychomotor retardation, and suicidal ideation. Anxious symptoms were measured with the Generalized Anxiety Disorder Scale [GAD-7; (44)], assessing if participants felt anxious, nervous, or on edge; experienced rumination, heightened tension or heightened irritability. For both questionnaires, the answering scale was 0 – *not at all*; 1 – *several days*; 2 – *more than half the days*; 3 – *nearly every day*. Symptoms of post-traumatic stress were measured with the Posttraumatic Stress Scale [PTSS-10; (45)], assessing the severity of sleep problems and nightmares, feelings of depression, jumpiness, wishes to withdraw from social interactions, irritability, mood swings, feelings of guilt, fear of triggering situations, and muscle tension. The answering scale ranged from 1 – *never* to 7 – *always*, with no further words describing the numbers 2–6.

#### 2.2.3 Measures to assess mental health impairments through PSYCA

To capture the psychopathological aspects of PSYCA, the mental health questionnaires assessing participants for symptoms of depression (PHQ-9), anxiety (GAD-7), and traumatic stress (PTSS-10), were presented a second time directly under the original item with the specifier ‘when thinking about climate change’. Thus, the first two items of the GAD-7 questionnaire were (1a) “Feeling nervous, anxious or on edge” and (1b) “… when thinking about climate change”; of the PHQ-9, (1a) “Little interest or pleasure in doing things” and (1b) “… when thinking about climate change”; of the PTSS-10, (1a) “sleep problems” and (1b) “when thinking about climate change.” By employing validated questionnaires offering a defined cut-off for clinically meaningful symptom load, we were able to assess the extent to which the emotions and mental states related to PSYCA represent a mental health impairment which is relevant in a psychopathological sense. Subsequently, the climate change-related item version of those questionnaires (e.g., “… when thinking about climate change”) will be referred to as the climate change versions of the mental health questionnaires: PHQ-9-C, GAD-7-C, and PTSS-10-C. As demonstrated in a previous study (30), the climate change versions of the questionnaires are clinically valid and show good to excellent internal consistencies.

#### 2.2.4 Measures to assess personality factors

For the identification of potential resilience factors, three questionnaires were used as measures for structural abilities (OPD-SF), sense of coherence (SOC-13), and attachment style (RQ). The short version of the OPD Structure Questionnaire (OPD-SF) was developed by Ehrenthal et al. (46) to capture the assessment of structural abilities according to OPD-2, previously only accessible through OPD (Operationalized Psychodynamic Diagnostics) interviews. The SOC scale captures the concept of a sense of coherence, which is regarded as a coping resource that makes people resilient to stressors and thus helps to promote health (47). It consists of three dimensions, namely comprehensibility, manageability and meaning (47). The German short version SOC-13 used here has a high internal consistency and correlates strongly with the long version of the scale (48). The RQ is a four-item measure, designed to assess attachment style (49). This study used a German translation and modification (50). Two sub-scores are derived, namely a robust attachment style with oneself (RQ-Self) and a robust attachment style with others (RQ-Other).

### 2.3 Statistical analysis

Data were analyzed using the software R (51). Descriptive statistics were generated to describe participants’ demographics, clinical symptom load, and their attitudes toward climate change. Regarding our guiding research questions, we (i) assessed whether persons with pre-existing mental health diagnoses show a quantifiable amount of psychopathology due to PSYCA by categorizing the climate change versions of the mental health questionnaires according to the validated cut-off scores of the questionnaires. We added an overview of the items per questionnaire that had been indicated to be the most impairing ones. Additionally, Cronbach’s alpha was calculated to test for internal consistency. To explore its relationship with non-specific ‘worry’ about climate change, we calculated correlation coefficients for the scores with the reported worry about climate change of the European Social Survey. To answer the question if (ii) psychopathological symptoms of depression, anxiety, and post-traumatic stress predict PSYCA in persons with pre-existing mental health diagnoses, we ran a logistical regression including the original versions of the mental health questionnaires, personality factors, age and gender. For the dependent variable (DV), participants were divided into those that reported at least some psychopathological symptoms of PSYCA and those who did not. Furthermore, we ran a multiple linear regression for an overall score of the climate change related versions of the mental health questionnaires (PHQ-9, GAD-7, PTSS-10) to assess if psychopathological symptoms of depression, anxiety, and post-traumatic stress predicted participants’ scores of psychopathological symptoms of PSYCA. Finally, to assess whether (iii) personality factors or demographical factors have an influence on participants’ scores of PSYCA, we added personality factors as a second block and age and gender as a third block to the multiple linear regression analyses.

## 3 Results

### 3.1 Descriptive statistics

In preparation for further analyses, data was screened for missing data and outliers. Of all questionnaires, 0.39% of data was missing. Due to the small percentage, we waived multiple imputation methods. However, as scores on all questionnaires were derived as sum scores, participants would have to be excluded listwise in case of one missing item. As missing data was evenly distributed, this would have led to the exclusion of several cases per analysis. Therefore, we calculated centered mean scores for all questionnaires, which would include cases with single missing items, as well, and based all subsequent analyses on those scores. Regarding outlier detection, we tested for univariate outliers by calculating the median absolute deviation and for multivariate outliers calculating the Mahalanobis distance, following the recommendations by Kline (52). Four cases were excluded as univariate outliers and one case as a multivariate outlier. Thus, all analyses were run for the remaining *n* = 84 participants.

Table 1 depicts the demographical characteristics of the participating patients (*n = 84*). Diagnoses could only be retrieved for *n* = 74 due to missing data. Patients were categorized into depressive disorders (F3X.XX), anxiety disorders (F40.XX, F41.XX), and post-traumatic stress disorder (F43.1). Aside from a depressive, anxiety, or post-traumatic stress disorder, 11 (13%) participating patients were diagnosed with an additional mental disorder (e.g., an eating disorder), 17 (20%) with two of the three disorders (depressive/anxious/post-traumatic), and 4 (5%) with all three disorders (depressive/anxious/post-traumatic). As a consequence, only four participants were diagnosed solely with an anxiety disorder, and only three participants solely with a post-traumatic stress disorder. Thus, we waived the possibility of exploring diagnosis-specific group differences.

**Table 1:** Demographical characteristics and mental health diagnoses of *n* = 84 psychosomatic outpatients.

Item	Specification	n	%
Sex	Male	26	31%
	Female	57	68%
	Diverse	1	1%
Age	M/SD	37.64	15.11
Education	Lower Secondary Education	6	7%
	Upper Secondary Education	38	45%
	Tertiary Education	36	43%
	Other	4	5%
Currently in psychotherapy	Yes	56	67%
	No	28	33%
Previously in psychotherapy	Yes, once	34	41%
	Yes, multiple times	32	38%
	No	18	21%
Currently taking psychotropic medication	Yes	39	46%
	No	45	54%
Diagnosis^1^	Depression	56	67%
	Anxiety Disorder	19	23%
	Post-Traumatic Stress Disorder	14	17%
	Missing	10	12%

^1^Multiple diagnoses were possible, hence the percentages add up to > 100%.

### 3.2 Attitudes toward climate change

99% of the sample stated that the climate is changing (88%) or probably changing (11%), only one person (1%) stated that the climate is probably not changing. Climate change mattered to most participants, as 69% stated to think about it very often or often and 70% stated they were extremely worried or very worried about it. Worries about climate change became more prevalent within the last years: 82% of the sample stated to worry more about climate change today than 5 years ago, 77% today more than 3 years ago and 55% reported to worry more today than last year. On a scale ranging from 0 (“not at all”) to 10 (“absolutely”), participants felt that a societal effort was the likelier scenario to mitigate climate change (M = 6.61, SD = 2.41) in comparison to an individual effort (M = 5.94, SD = 2.12). Nevertheless, participants did not think it to be likely that enough countries would put measures into place that are in time to effectually mitigate climate change (M = 3.90, SD = 2.02).

### 3.3 Overall and climate change related psychopathology

Patients’ sum scores on the mental health questionnaires for clinical and climate change related mental health burden were transformed into clinically meaningful categories relying on the standard samples reported in the respective manuals or publications. Translated scores are displayed in Table 2. GAD-7, PHQ-9, and PTSS-10 showed good internal consistencies, with Cronbach’s alpha α = 0.82 for all three questionnaires. The climate change related versions showed good internal consistencies, with α = 0.82 for PHQ-9-C, α = 0.83 for GAD-7-C, and α = 0.82 for PTSS-10-C. Overall, participants reported higher levels of clinical symptom load than for symptom load regarding climate change awareness. For symptom load regarding climate change awareness, the highest percentage of symptoms was reported for the GAD-7-C. Means, standard deviations, minimum and maximum scores of the original sum scores of all questionnaires are displayed in Table 3.

**Table 2:** Participants’ clinical symptom load and symptom load regarding climate change awareness.

Mental health disorder (Measure)	Original version		Modified climate change related version	
	*n*	%	*n*	%
Depressive disorder (PHQ-9; PHQ-9-C)^a^				
No symptom burden	7	8%	73	87%
Mild symptoms	20	24%	9	11%
Moderate symptoms	24	29%	-	–
Severe symptoms	31	37%	-	–
Missing	2	2%	2	2%
General anxiety disorder (GAD-7; GAD-7-C)^b^				
No symptom burden	8	10%	64	76%
Low levels of anxiety	24	29%	17	20%
Moderate levels of anxiety	34	40%	1	1%
High levels of anxiety	18	21%	-	–
Missing	-	–	2	2%
Traumatic symptom burden (PTSS-10; PTSS-10-C)^c^				
No significant traumatic symptom burden	25	30%	83	99%
Positively screened for traumatic symptom burden	58	69%	-	–
Missing	1	1%	1	1%

Annotation. ^a^Brief Patient Health Questionnaire (PHQ-9, PHQ-9-C) cut-offs: 5–9 = mild symptoms; 10–14 = moderate symptoms; ≥15 = severe symptoms. ^b^Generalized Anxiety Disorder Scale (GAD-7, GAD-7-C) cut-offs: 5–9 = low levels of anxiety; 10–14 = moderate levels of anxiety; ≥15 = high levels of anxiety. ^c^Posttraumatic stress scale (PTSS-10, PTSS-10-C) cut-off: ≥35 = suspected traumatic symptom burden.

**Table 3:** Mean, standard deviation and range of the raw scores of participants’ clinical symptom load and symptom load regarding climate change awareness.

Mental health measures	*n*	*M*	*SD*	*Min*	*Max*
PHQ-9^a^	82	12.99	6.24	0	25
PHQ-9-C^a^	82	1.51	1.93	0	7
GAD-7^b^	84	10.82	4.70	0	21
GAD-7-C^b^	82	2.60	2.61	0	12
PTSS-10^c^	83	40.43	12.43	10	64
PTSS-10-C^c^	83	14.81	5.56	10	32

Annotation. ^a^Brief Patient Health Questionnaire; ^b^Generalized Anxiety Disorder Scale; ^c^Posttraumatic stress scale.

**Figure 1:**
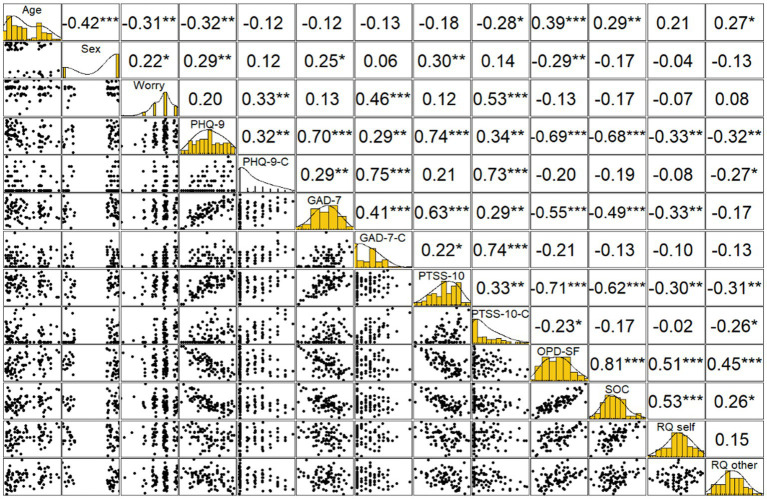
Correlation coefficients, plots, and histograms of data distribution for worry, general depressive symptoms (PHQ-9), climate change awareness related depressive symptoms (PHQ-9-C), general anxious symptoms (GAD-7), climate change awareness related anxious symptoms (GAD-7-C), general post-traumatic symptoms (PTSS-10), climate change awareness related post-traumatic symptoms (PTSS-10-C), personality functioning (OPD-SF), sense of coherence (SOC), relationship style toward self (RQ-Self), relationship-style toward other (RQ-Other).

Correlation coefficients, plots, and histograms of data distribution, including personality factors and worry about climate change, are displayed in Figure 1. Scores on the OPD-SF were reversed for better interpretability, hence a high score indicates a high level of structural integration. As tested for with the Shapiro–Wilk test, data for the climate change related questionnaires was not normally distributed. Thus, correlation coefficients were calculated using Spearman rank-order correlation as a conservative estimator eliminating possible inflation of correlation coefficients in case of non-normality (53).

To better understand which items of the questionnaires employed in our study described symptoms that participants experienced regarding PSYCA, we tested which items were rated most often. To achieve this, we ordered the items by the total amount of symptom load participants had indicated for those items, e.g., in case of GAD-7-C, the sum of all 84 ratings ranging from 0 to 3. The most rated items of PHQ-9-C were feeling down, depressed, or hopeless; a decrease of interest or pleasure in doing things; and the feeling to be a failure and to disappoint. In the GAD-7-C, the most rated items were excessive worrying; feeling scared, like something bad is about to happen; and feeling anxious, nervous, or on edge. In the PTSS-10-C, the most rated items were feelings of guilt; feeling depressed; and irritability.

### 3.4 Logistic regression predicting which participants experience PSYCA

Logistic regression was used to analyze the relationship of PSYCA with overall psychopathology. To this end, participants were divided into a group reporting no symptoms of PSYCA at all (*n* = 21) and a group reporting at least some symptoms (*n* = 61), with two participants excluded because division was based on sum scores and single items were missing in two cases. Personality factors, age and gender, and mental health diagnoses were added block wise to test for additional explanatory value. The model including overall psychopathology and mental health diagnoses showed the lowest AIC. Thus, this model was retained and can be seen in Table 4. Out of all predictors tested for, the score of overall anxiety (GAD-7) was the only significant predictor, *OR* = 4.96 (95% CI [1.19–23.80]), when holding all other factors constant.

**Table 4:** Logistic regression to predict which participants (*n* = 82) reported at least some symptoms of PSYCA, with overall psychopathology and psychiatric diagnoses as predictors.

Variable				95% CI		
	*Estimate*	*E*	*OR^b^*	*lower*	*upper*	*p*
Intercept	0.01	0.60	1.01	0.30	3.34	0.982
*PHQ-9*	0.97	0.76	2.65	0.62	13.04	0.200
*GAD-7*	1.60	0.75	4.98	1.19	23.80	0.033
*PTSS-10*	−0.73	0.44	0.48	0.19	1.12	0.100
*Diagnosis: anxiety*	0.59	0.77	1.81	0.43	9.69	0.444
*Diagnosis: depression*	1.20	0.79	3.34	0.82	14.57	0.095
*Diagnosis: PTSD^a^*	−0.23	0.79	0.79	0.17	4.07	0.776

Annotations. ^a^Post-Traumatic Stress Disorder; ^b^Odds Ratio.

### 3.5 Multiple linear regressions predicting the experienced extent of PSYCA

To further examine the relationship pf PSYCA with the other variables, multiple regression analyses were run for each of the questionnaires, PHQ-9-C, GAD-7-C, and PTSS-10-C. Participants reporting at least some symptoms (*n* = 61) were included in the analysis. In a first model, the remaining two climate change related questionnaires (in case of PHQ-9-C, for example, GAD-7-C and PTSS-10-C) were entered as predictors. Afterward, overall scores of depressive, anxious, and traumatic symptoms (PHA-9, GAD-7, PTSS-10) were added in one block, personality factors as an additional block, and age, gender, and the corresponding mental health diagnosis (e.g., in case of PHQ-9-C, a diagnosis of depression) as the final block. If the inclusion of a block increased the amount of explained variance as indicated by *R^2^*, the block was kept and the next block was added. The final models per questionnaire are displayed in Table 5. Normality of residuals, normality of random effects, linear relationship, homogeneity of variance, and multicollinearity were acceptable for all models and the respective analyses can be found in appendix 1.

**Table 5:** Multiple regressions predicting climate change awareness related symptoms of depression, anxiety, and post-traumatic stress in *n* = 61 patients of a psychosomatic outpatient clinic.

	PHQ-9-C						
Variable								95% CI
	B	*β*	SE_B_	*t*	*p*	Lower	Upper
Intercept	−0.04	−0.01	0.03	−1.24	0.221	−0.093	0.022
GAD-7-C	0.25	0.40	0.07	3.43	0.001	0.103	0.395
PTSS-10-C	0.16	0.40	0.05	3.21	0.002	0.059	0.254
PHQ-9	0.13	0.37	0.04	2.89	0.005	0.040	0.220
GAD-7	−0.03	0.09	0.04	−0.76	0.453	−0.124	0.056
PTSS-10	−0.04	0.17	0.03	−1.26	0.213	−0.095	0.022
*R^2^* = 0.583, *R^2^_adj_* = 0.545							
								GAD-7-C
Intercept	0.06	−0.01	0.05	1.29	0.201	−0.034	0.158
PHQ-9-C	0.71	0.44	0.21	3.43	0.001	0.293	1.119
PTSS-10-C	0.24	0.39	0.08	2.81	0.007	0.067	0.402
GAD-7	0.21	0.37	0.07	3.04	0.004	0.073	0.354
PHQ-9	−0.09	−0.16	0.08	−1.07	0.288	−0.247	0.075
PTSS-10	−0.07	−0.22	0.05	−1.46	0.149	−0.168	0.026
*R^2^* = 0.609, *R^2^_adj_* = 0.539							
								PTSS-10-C
Intercept	0.39	−0.01	0.31	1.25	0.218	−0.239	1.022
PHQ-9-C	1.00	0.38	0.35	2.89	0.006	0.305	1.703
GAD-7-C	0.47	0.29	0.20	2.33	0.024	0.065	0.880
PTSS-10	0.26	0.48	0.08	3.32	0.002	0.102	0.416
PHQ-9	−0.23	−0.25	0.15	−1.55	0.129	−0.521	0.068
GAD-7	−0.12	−0.12	0.13	−0.98	0.334	−0.375	0.130
Diag. PTSD	0.18	0.12	0.16	1.15	0.255	−0.135	0.496
Age	−0.01	−0.21	0.004	−2.04	0.047	−0.017	0.000
Gender	−0.07	−0.06	0.13	−0.53	0.597	−0.331	0.193

*R^2^* = 0.648, *R^2^_adj_* = 0.586.

The final models explained between 54% and 59% of the variance in the criterion variables, as indicated by adjusted *R^2^*. For all three questionnaires, higher scores on the clinical symptom questionnaires (PHQ-9 for PHQ-9-C, GAD-7 for GAD-7-C, PTSS-10 for PTSS-10-C) predicted higher scores for the same diagnostic entities regarding PSYCA, and higher scores on the climate change related versions of the mental health questionnaires predicted each other. There were no additional significant predictors for the PHQ-9-C. For the GAD-7-C, a high level of structural integration (OPD-SF) was a significant predictor for higher scores on the GAD-7-C (*β* = − 0.42, *p* = 0.021), and higher scores on the sense of coherence scale (SOC-13) were a predictor for lower scores on the GAD-7-C (*β* = 0.39, *p* = 0.025). However, as the positive correlation in case of the OPD-SF seemed counter-intuitive, and the two predictors showed a high collinearity in a post-hoc analysis, we ran the analysis again with only one of the predictors remaining in the model. This time, both predictors were not significant, confirming our hypothesis of an artificial suppression effect (54). Therefore, we did not include them in our final model. Finally, younger age was a significant predictor for higher scores on the PTSS-10-C (*β* = − 0.21, *p* = 0.047).

## 4 Discussion

In the present study, we explored mental health impairments through PSYCA in persons with pre-existing mental health diagnoses and its relationship with personality factors and demographical factors. As the awareness for climate change is a prerequisite to feel distressed about it, we included questions about participants’ perception of climate change into our study. In line with the findings in the original study (42), virtually all participants (99%) stated that the climate is changing. A majority reported to be worried or extremely worried about it, and those worries have increased over the last years. In our study, we were able to contrast this high percentage of people reporting undifferentiated ‘worry’ with the number of people experiencing psychopathological symptoms to a clinically meaningful extent. Out of all participants, 11% reported mild depressive symptoms due to PSYCA, and 20% mild anxious symptoms due to PSYCA (one person [1%] reporting moderate levels of anxiety), and no participant reported traumatic symptoms due to PSYCA. The reported extent of worry correlated significantly with all PSYCA symptom questionnaires. Hence, in line with previous research (9, 21), worries did not translate directly into clinical mental health burden due to PSYCA, but there seems to be a linear relationship between the constructs. Framed in a diathesis-stress model (55), worries about climate change could be perceived as one, but not the single decisive factor for mental health impairments due to PSYCA.

To identify additional contributing factors, we ran a logistic regression predicting which participants reported at least some symptoms of PSYCA. General anxiety as measured with the GAD-7 turned out to be the only significant predictor, indicating that a generally anxious state makes it likelier to be impaired by anxieties concerned with climate change. However, as outlined in a similar study reporting symptoms of generalized anxiety to predict climate change anxiety (56), psychosomatic outpatients could possibly perceive every further burdening topic as a greater mental health burden than persons without a pre-existing mental health diagnosis. Further research comparing patients’ anxious reactions to climate change with their reaction to other topics, such as an economic crisis or a pandemic, is warranted. This might help to understand if mental health impairments due to PSYCA are a distinct phenomenon or rather an expression of an underlying vulnerability toward any potentially worrisome topic.

Nonetheless, the number of psychosomatic outpatients reporting anxious or depressive symptoms due to PSYCA are considerably higher than in a group of medical students that we assessed during the same period of time (30), and higher than the percentages in a recent population-based poll in the US which employed the short version of the GAD-7 and the PHQ-9, the PHQ-4 (29). Hence, mental health impairments due to PSYCA may not (as yet) be a primary motive for the majority of people to seek psychotherapy, but it may contribute to patients’ symptomology. Psychotherapists should therefore consider integrating a discussion of the topic into their anamnesis, and be aware that climate change related topics might contribute to patients’ mental health burden. A recent qualitative analysis of patients’ perceptions of helpful therapist behaviors to cope with the mental health burden regarding climate change awareness suggests that therapists should have a basic knowledge about climate change and its consequences, provide an existential perspective on the topic, and offer a discussion of possible ways to cope with the adverse psychological effects (13).

The significant share of variance explained by the other two climate change related mental health questionnaires (e.g., in case of the PHQ-9-C, the GAD-7-C and the PTSS-10-C) may reflect the multifactorial nature of PSYCA. In addition to these predictors, PSYCA was predicted by overall symptoms for the same diagnostic entities, but not of the other two diagnostic entities that had been assessed. Therefore, understanding PSYCA as a superordinate construct which shows itself predominantly in the form of symptoms the person is already experiencing seems plausible. This may be due to dysfunctional cognitive processes contributing to the development and perpetuation of mental health impairments. Affective experience influences everyday perception and cognition (57). In persons with diagnosed depressive, anxious or post-traumatic stress disorders, biased attentional and memory processes, as well as mood congruent processing of information, affects the onset, maintenance and course of mental health impairments (58–60). Therefore, psychosomatic outpatients reporting more depressive symptoms might focus more on the sadness-related feelings evoked by PSYCA, like grief or meaninglessness, whereas those reporting more anxious or post-traumatic symptoms might rather focus on the threat-related feelings evoked by PSYCA, like anxiety or shock. As a consequence, the same cognitive biases contributing to clinical symptoms of depression, anxiety, or post-traumatic stress, might contribute to the development and perpetuation of the same PSYCA symptoms.

Of note, personality factors did not explain a significant share of variance in PSYCA. This may be a consequence of the relatively small number of participants reporting those symptoms, resulting in a restrained variance for the multiple regression analyses, as those factors did correlate significantly with the scores of GAD-7-C, PHQ-9-C, and PTSS-10-C in single correlational analysis (see Figure 1). Furthermore, all constructs employed in our study (structural abilities, sense of coherence, and attachment style) are understood as stable, trait-like factors. The missing contribution in explaining the shared variance of PSYCA could possibly be grounded in a more state-like nature of PSYCA, covarying more closely with other state-like constructs, such as psychiatric symptoms. When selecting these questionnaires for our study, we were driven by the idea that people might display some stable attributes which could be linked to PSYCA; so far, we have not been able to verify such an understanding, and research into other possible covariates of PSYCA is warranted. Considering demographical factors, only the score of traumatic symptoms due to PSYCA was negatively predicted by age. Again, this result has to be viewed with caution, as no participant reported a clinically significant score of traumatic symptoms due to PSYCA. The lack of influence of age and gender on PSYCA, in combination with the much higher number of persons reporting symptoms in comparison to the general population, might indicate that a pre-existing mental health diagnosis is a more severe risk factor for the development of PSYCA than young age or female sex.

### 4.1 Strengths and limitations

To the best of our knowledge, the present study is the first to explore the extent of psychopathology due to PSYCA in persons with pre-existing mental health diagnoses and its association with general clinical symptomology. However, several limitations should be considered. Firstly, the sample in the present study was a convenience sample and included only patients in an outpatient setting, and was limited in its size, thus generalizability to a general population of persons with mental health diagnoses might be limited. Furthermore, participation was voluntary, and therefore participants with an interest in the psychological effects of climate change might be overrepresented in comparison to a representative population sample. The adapted versions of the questionnaires used to assess psychological symptoms due to PSYCA have not been developed or validated for this use. However, as the items are a description of psychological or bodily symptoms that are inherent to the respective disorder, an assessment of symptom severity can be derived from the scores, regardless of their cause. As the items assessing general symptom load and symptom load regarding PSYCA were presented in direct succession, a recall bias might be expected. However, the substantive differences in symptom severity reported by participants when reporting anxious, depressive or post-traumatic symptoms in general or regarding PSYCA and the only moderate correlations of the general and PSYCA-related scales per construct (*r* = 0.32–0.41; see Figure 1) are indicating that participants were able to distinguish between general and PSYCA-related symptoms. Regarding the association of personality factors and climate change awareness related mental health burden, it is important to consider the cross-sectional nature of the data presented and not to draw any causal conclusions.

## 5 Conclusion

The heightened vulnerability of persons with pre-existing mental health diagnoses to the adverse psychological effects of climate change, with a substantial part of the present sample already reporting mild or moderate symptoms, solicits an adequate response from the health care system. Further research on the definition, development and association with mental disorders is needed; protective factors to foster resilience have to be identified; and knowledge about treatment options, especially in psychotherapy, has to be broadened. The concept of climate change awareness as an impairment to mental wellbeing is still in development, and considering its growing effect on an individual and societal level, its understanding and treatment should be developed accordingly.

## Data availability statement

The data presented in this article are not readily available because of legal restrictions placed on patient data in Germany. Requests to access the data should be directed to nadja.gebhardt@med.uni-heidelberg.de and will be granted upon reasonable request.

## Ethics statement

The studies involving humans were approved by Ethics committee of the Medical Faculty of the University of Heidelberg. The studies were conducted in accordance with the local legislation and institutional requirements. The participants provided their written informed consent to participate in this study.

## Author contributions

NG: Conceptualization, Data curation, Formal analysis, Methodology, Visualization, Writing – original draft, Writing – review & editing. LS: Conceptualization, Data curation, Investigation, Project administration, Writing – review & editing. H-CF: Funding acquisition, Resources, Software, Supervision, Writing – review & editing. CN: Conceptualization, Funding acquisition, Project administration, Resources, Software, Supervision, Validation, Writing – review & editing.
