# Supplementary material for: The relationship of climate change awareness and psychopathology in persons with pre-existing mental health diagnoses
Source: Front Psychiatry. 2023 Nov 27;14:1274523. doi: 10.3389/fpsyt.2023.1274523 (PMC10715411; doi:10.3389/fpsyt.2023.1274523)
Supplement: Supplementary file 1 [file Data_Sheet_1.PDF]

Appendix 1: Normality of residuals, normality of random effects, linear relationship, homogeneity of variance, and multicollinearity of the presented multiple linear regression models predicting the experienced extent of PSYCA

PHQ-9-C

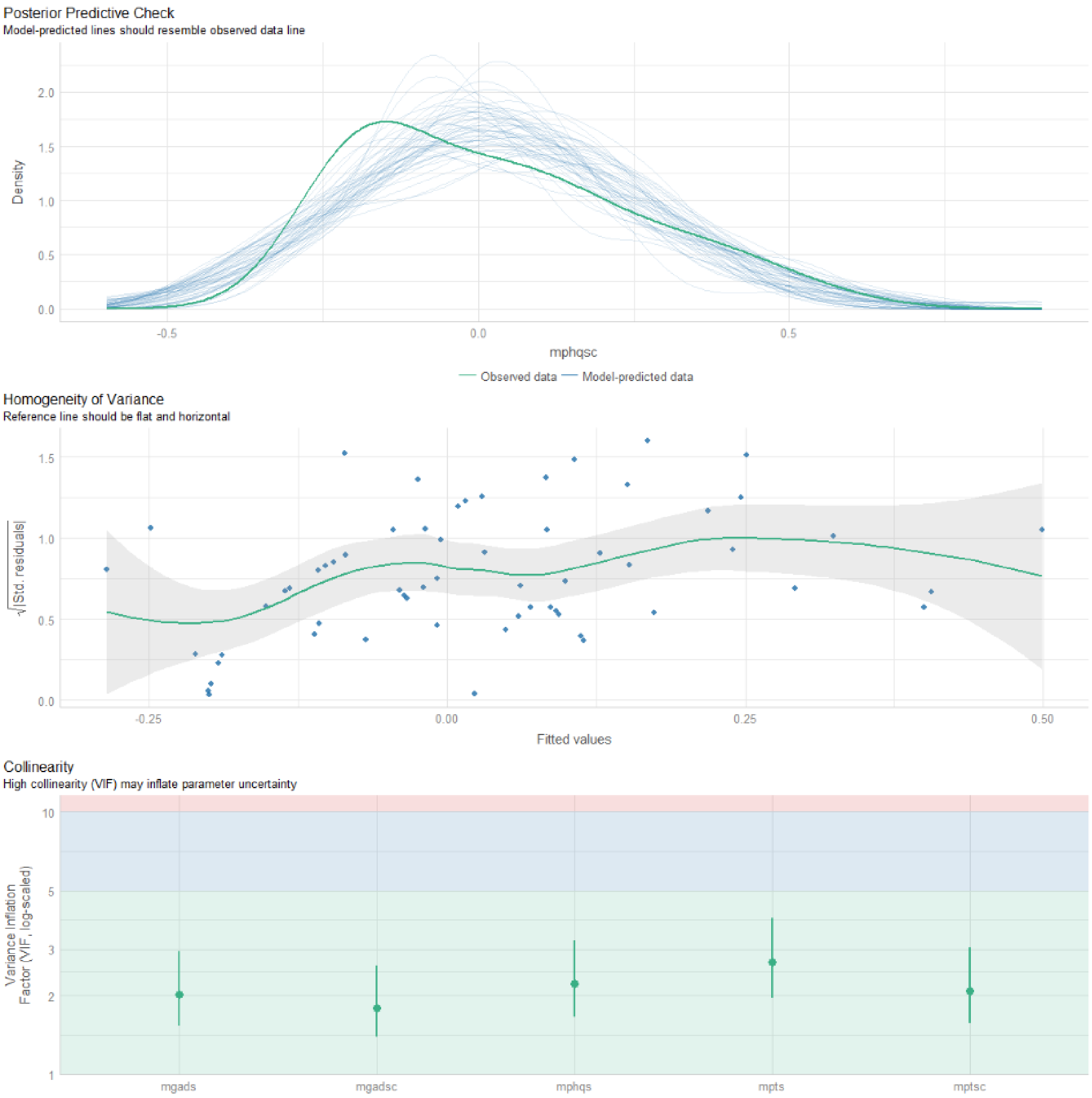

Linearity

Reference line should be flat and horizontal

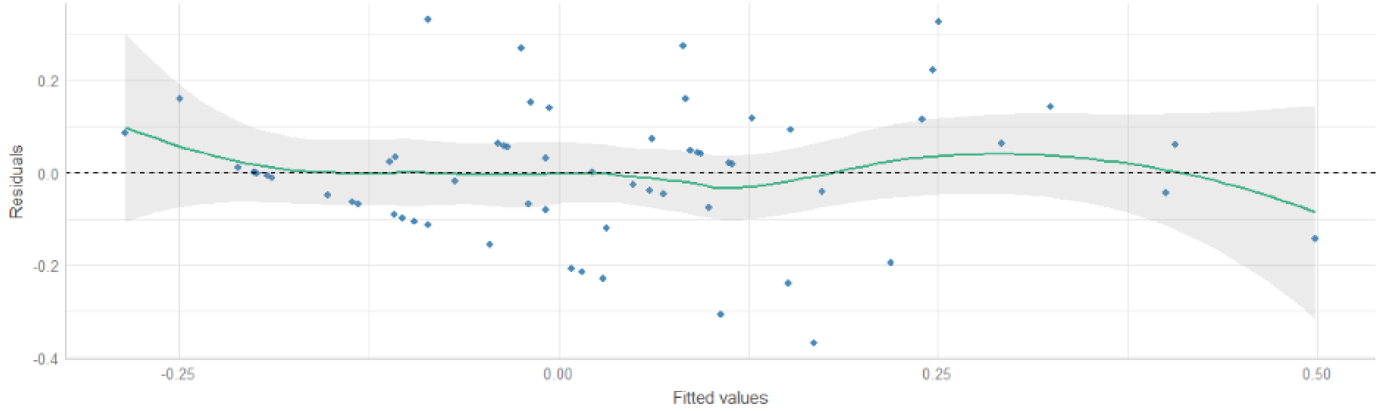

Influential Observations

Points should be inside the contour lines

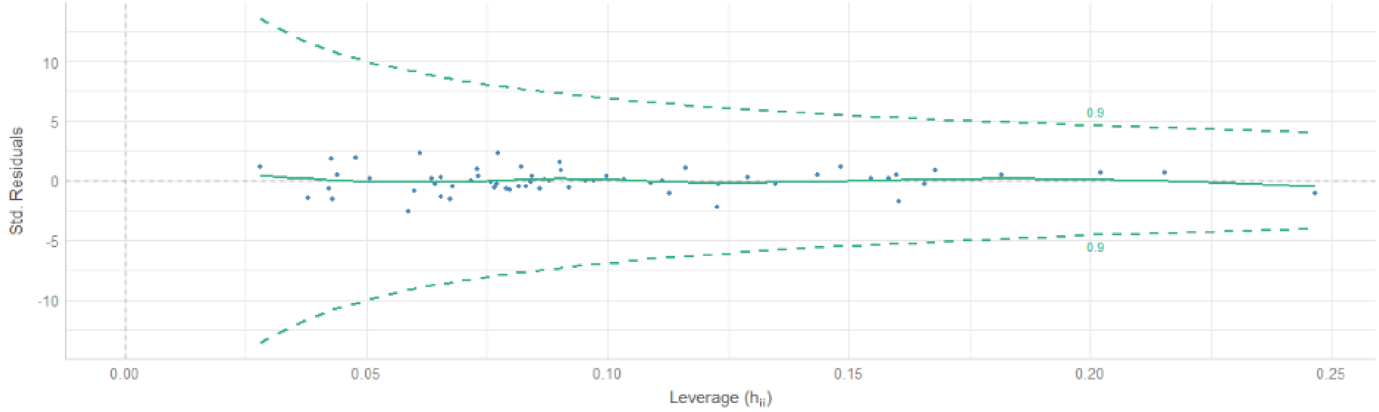

Normality of Residuals

Dots should fall along the line

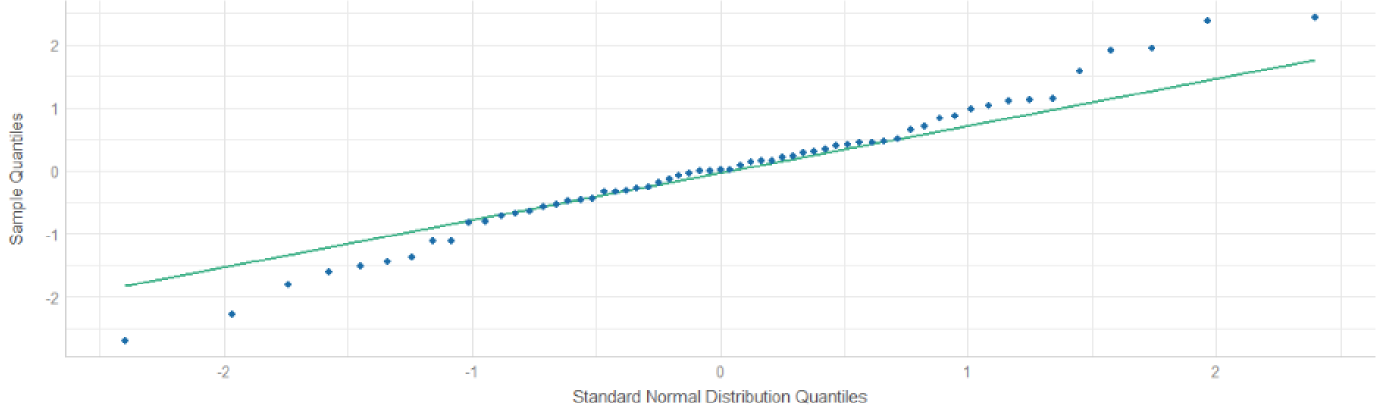

GAD-7-C

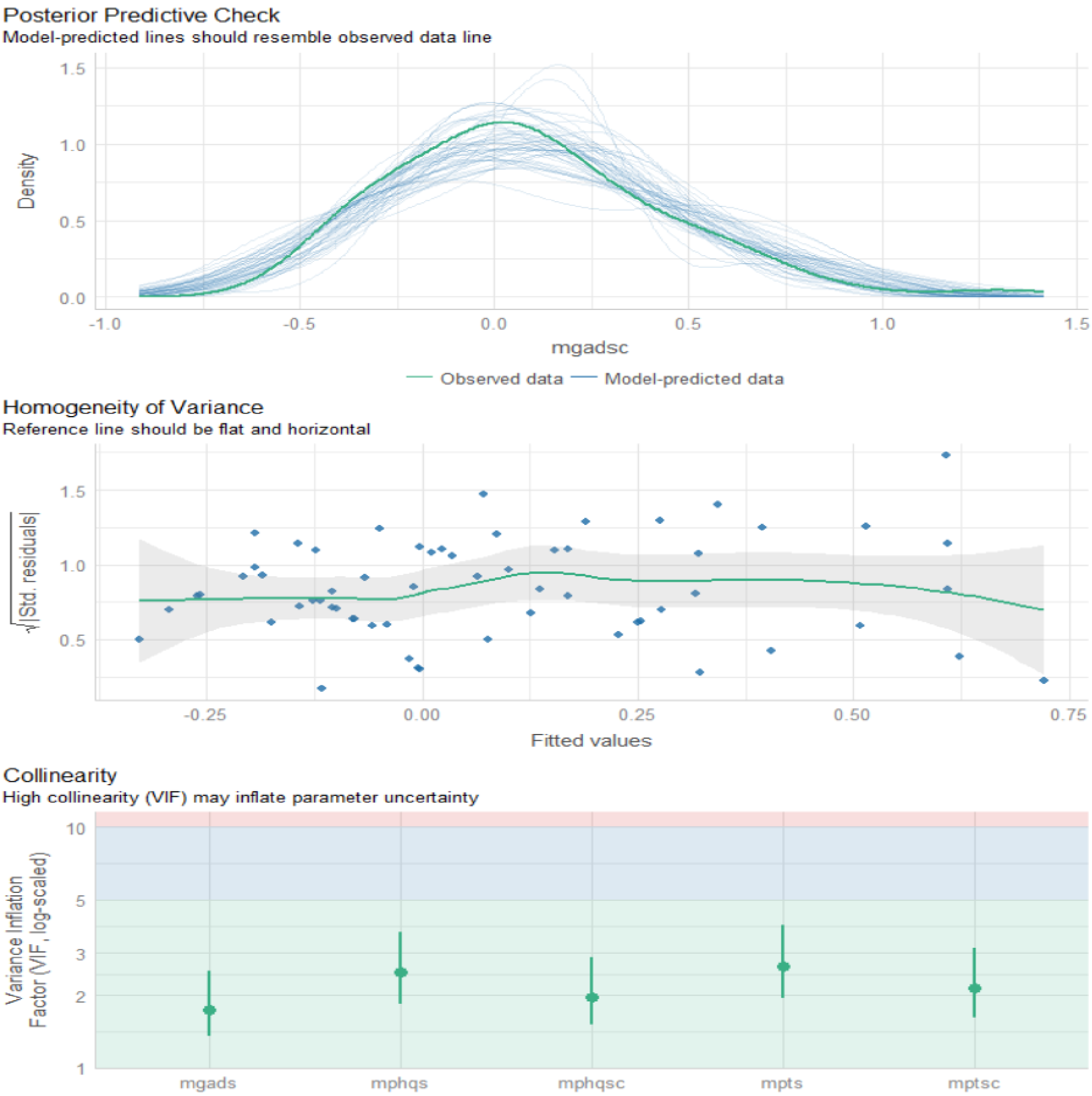
Posterior Predictive Check
Model-predicted lines should resemble observed data line
Density
1.5
1.0
0.5
0.0
-1.0
-0.5
0.0
0.5
1.0
1.5
mgadsc
Observed data
Model-predicted data
Homogeneity of Variance
Reference line should be flat and horizontal
√|Std. residuals|
1.5
1.0
0.5
-0.25
0.00
0.25
0.50
0.75
Fitted values
Collinearity
High collinearity (VIF) may inflate parameter uncertainty
Variance Inflation Factor (VIF, log-scaled)
10
5
3
2
1
mgads
mphqs
mphqsc
mpts
mptsc

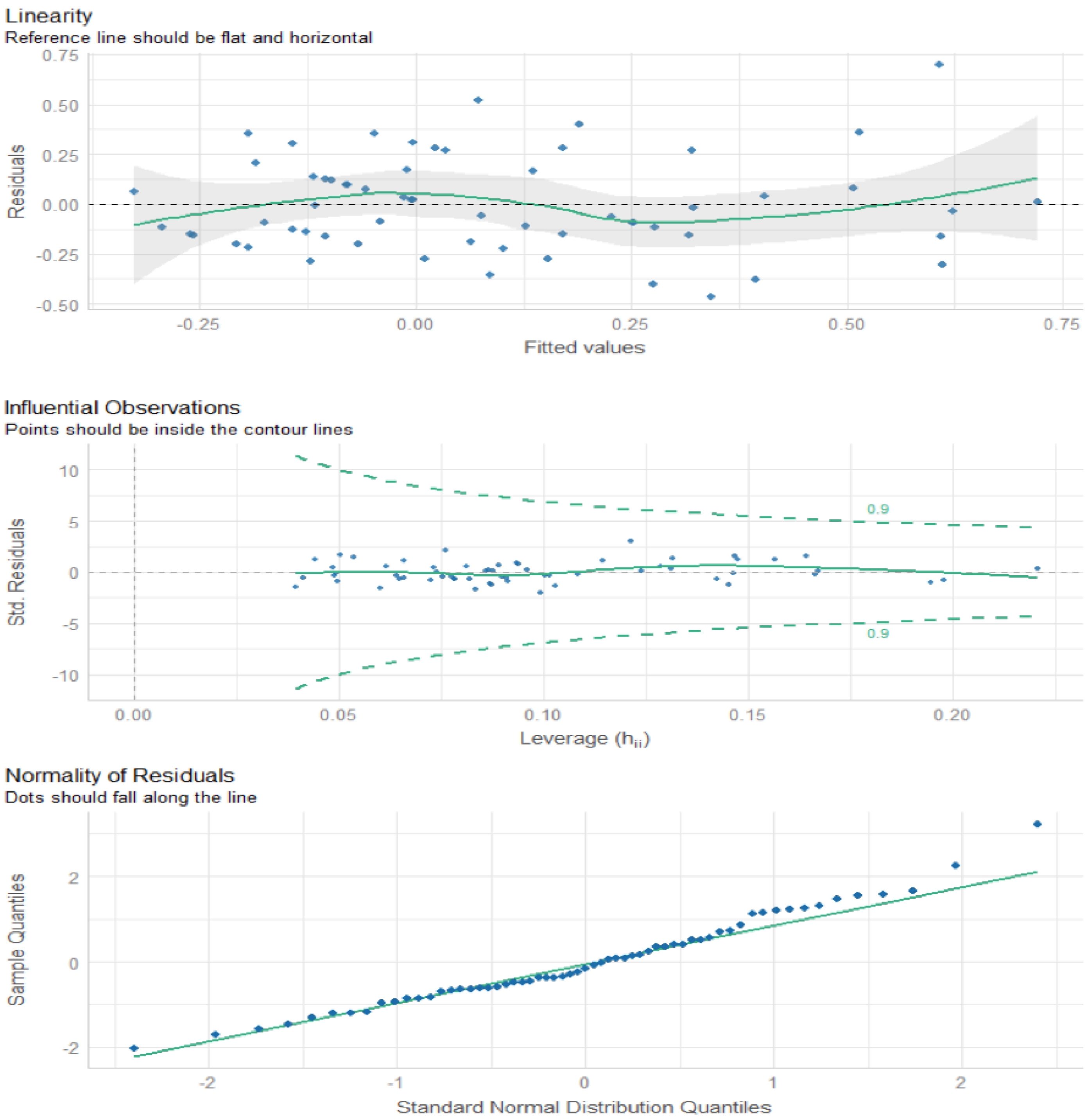
Linearity
Reference line should be flat and horizontal
Residuals
0.75
0.50
0.25
0.00
-0.25
-0.50
-0.25
0.00
0.25
0.50
0.75
Fitted values
Influential Observations
Points should be inside the contour lines
Std. Residuals
10
5
0
-5
-10
0.9
0.9
0.00
0.05
0.10
0.15
0.20
Leverage (h_{ii})
Normality of Residuals
Dots should fall along the line
Sample Quantiles
2
0
-2
-2
-1
0
1
2
Standard Normal Distribution Quantiles

PTSS-10-C

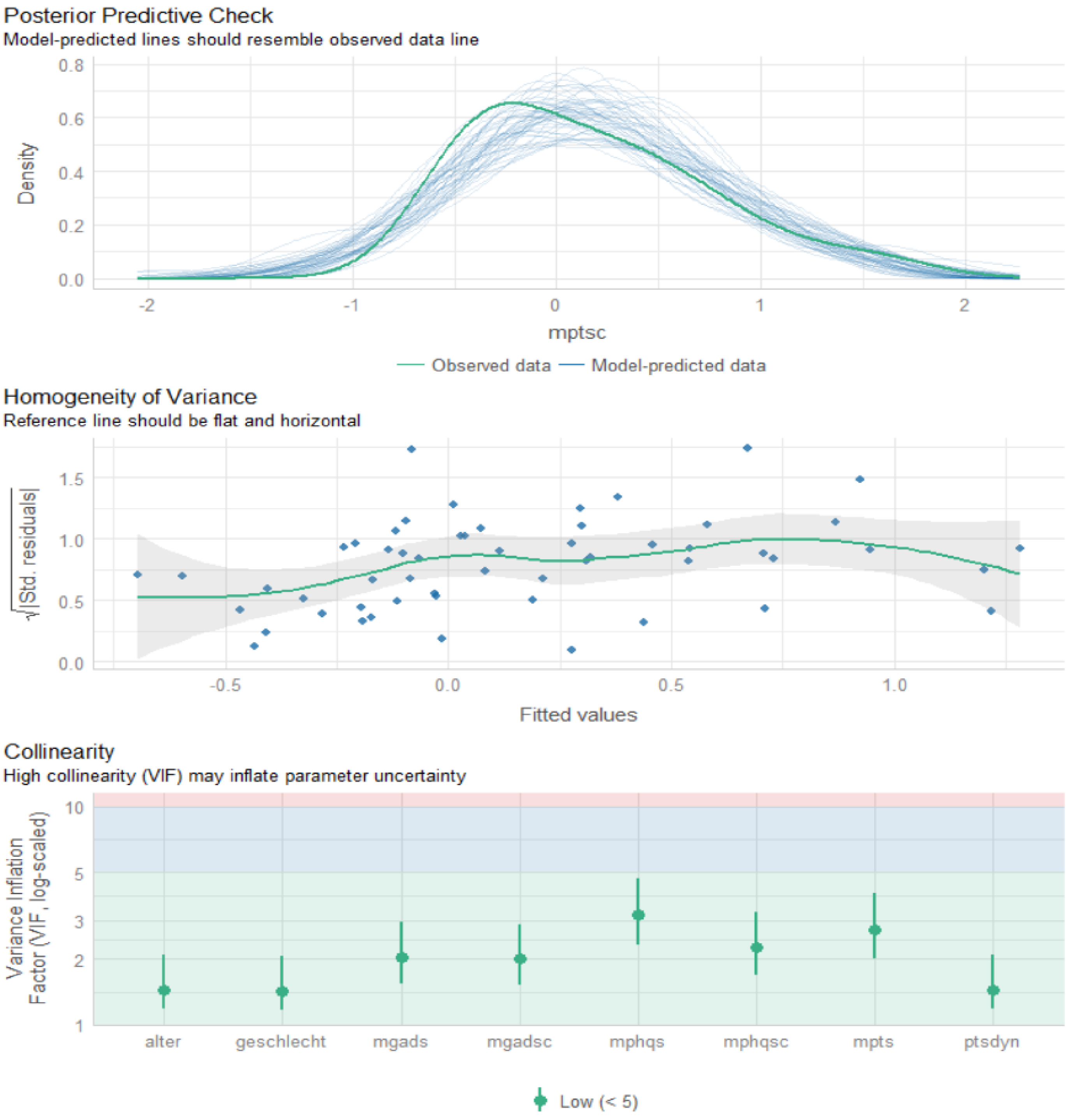
Posterior Predictive Check
Model-predicted lines should resemble observed data line
Density
0.8
0.6
0.4
0.2
0.0
-2
-1
0
1
2
mptsc
Observed data
Model-predicted data
Homogeneity of Variance
Reference line should be flat and horizontal
√|Std. residuals|
1.5
1.0
0.5
0.0
-0.5
0.0
0.5
1.0
Fitted values
Collinearity
High collinearity (VIF) may inflate parameter uncertainty
Variance Inflation Factor (VIF, log-scaled)
10
5
3
2
1
alter
geschlecht
mgads
mgadsc
mphqs
mphqsc
mpts
ptsdyn
Low (< 5)

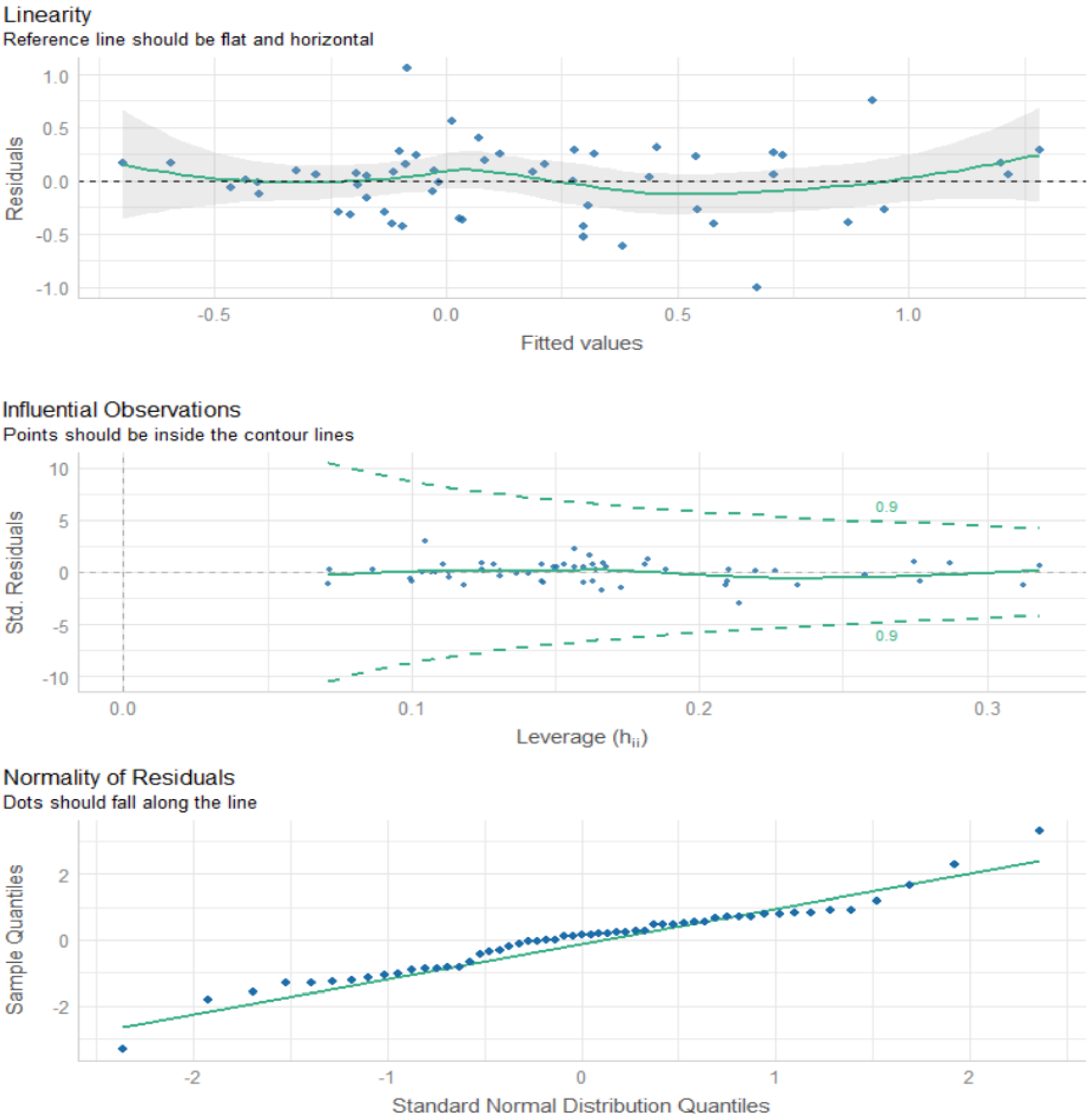
Linearity
Reference line should be flat and horizontal
Residuals
Fitted values
Influential Observations
Points should be inside the contour lines
Std. Residuals
Leverage (h_{ii})
0.9
Normality of Residuals
Dots should fall along the line
Sample Quantiles
Standard Normal Distribution Quantiles
